# 4,5-Dihydro-3a,5a-diazo­niapyrene triiodidocuprate(I)

**DOI:** 10.1107/S1600536811050136

**Published:** 2011-11-25

**Authors:** Di Wu, Peng-Chao Hao, Yun-Yin Niu, Seik Weng Ng, Edward R. T. Tiekink

**Affiliations:** a Department of Chemistry, Zhengzhou University, Zhengzhou, People’s Republic of China; c Department of Chemistry, University of Malaya, 50603 Kuala Lumpur, Malaysia; b Chemistry Department, Faculty of Science, King Abdulaziz University, PO Box 80203 Jeddah, Saudi Arabia

## Abstract

In the dianion of the title salt, (C_14_H_12_N_2_)[CuI_3_], the Cu^I^ atom is coordinated by three I^−^ ions that define a nearly trigonal-planar geometry; the Cu^I^ atom lies 0.1407 (6) Å out of the plane. With the exception of the methyl­ene C atoms, the dication is essentially planar (r.m.s deviation = 0.067 Å). The most significant inter­action between the ions is a C—H⋯I contact.

## Related literature

For studies of the triiodidocuprate(I) di-anion, see: Mishra *et al.* (2008 ▶); Su *et al.* (2003 ▶). For background to the phenanthrolinium di-cation as a template for the construction of thio­cyanato­cuprate(I) polymers, see: Yue *et al.* (2010 ▶). For information on the Cambridge Structural Database, see: Allen (2002 ▶).
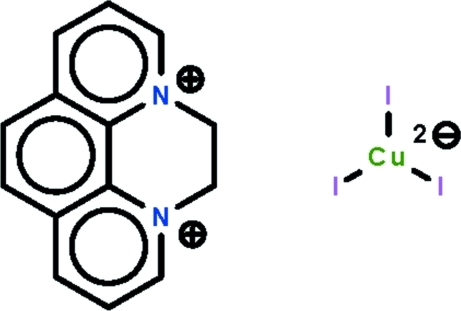


## Experimental

### 

#### Crystal data

(C_14_H_12_N_2_)[CuI_3_]
*M* *_r_* = 652.50
Monoclinic, 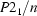

*a* = 7.6018 (6) Å
*b* = 15.0917 (12) Å
*c* = 14.2776 (12) Å
β = 98.903 (1)°
*V* = 1618.2 (2) Å^3^
*Z* = 4
Mo *K*α radiation
μ = 7.06 mm^−1^
*T* = 100 K
0.20 × 0.20 × 0.02 mm

#### Data collection

Bruker SMART APEX diffractometer
Absorption correction: multi-scan (*SADABS*; Sheldrick, 1996 ▶) *T*_min_ = 0.332, *T*_max_ = 0.872
14984 measured reflections
3701 independent reflections
3065 reflections with *I* > 2σ(*I*)
*R*_int_ = 0.055

#### Refinement

*R*[*F* ^2^ > 2σ(*F* ^2^)] = 0.028
*wR*(*F* ^2^) = 0.065
*S* = 1.05
3701 reflections
181 parameters
6 restraints
H-atom parameters constrained
Δρ_max_ = 0.73 e Å^−3^
Δρ_min_ = −0.82 e Å^−3^

### 

Data collection: *APEX2* (Bruker, 2009 ▶); cell refinement: *SAINT* (Bruker, 2009 ▶); data reduction: *SAINT*; program(s) used to solve structure: *SHELXS97* (Sheldrick, 2008 ▶); program(s) used to refine structure: *SHELXL97* (Sheldrick, 2008 ▶); molecular graphics: *X-SEED* (Barbour, 2001 ▶) and *DIAMOND* (Brandenburg, 2006 ▶); software used to prepare material for publication: *publCIF* (Westrip, 2010 ▶).

## Supplementary Material

Crystal structure: contains datablock(s) global, I. DOI: 10.1107/S1600536811050136/bt5714sup1.cif

Structure factors: contains datablock(s) I. DOI: 10.1107/S1600536811050136/bt5714Isup2.hkl

Additional supplementary materials:  crystallographic information; 3D view; checkCIF report

## Figures and Tables

**Table 1:** Selected bond lengths (Å)

Cu—I1	2.5336 (7)
Cu—I2	2.5254 (7)
Cu—I3	2.5025 (7)

**Table 2:** Hydrogen-bond geometry (Å, °)

*D*—H⋯*A*	*D*—H	H⋯*A*	*D*⋯*A*	*D*—H⋯*A*
C13—H13b⋯I2	0.99	3.06	3.969 (4)	154

**Fig. 1.**
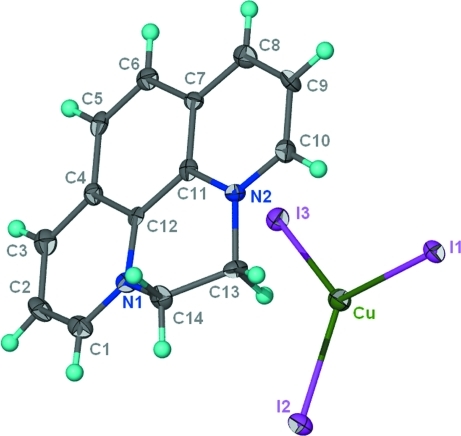
Displacement ellipsoid plot (Barbour, 2001) of (I) drawn at the 70% probability level; hydrogen atoms are drawn as spheres of arbitrary radius.

## supplementary crystallographic information

### Comment

Organic-inorganic hybrid compounds containing the triiodidocuprate(I) anion have been the subject of recent investigations (Mishra *et al.*, 2008; Su *et al.*, 2003). While the phenanthrolinium di-cation has proved to be a suitable template for the construction of a thiocyanatocuprate(I) polymer (Yue *et al.*, 2010), crystal structures containing the phenanthrolinium template are relatively scarce (Allen, 2002). In this communication the crystal structure of a new triiodidocuprate(I) complex containing the 5,6-dihydrodipyrazino(1,2,3,4-lmn)-1,10-phenanthrolinium dication, *i.e*. (I), is described.

The crystallographic asymmetric unit of (I), comprises a di-cation and a di-anion, Fig. 1. The 14 non-hydrogen atom comprising the aromatic part of the di-cation are effectively planar with a r.m.s. deviation = 0.067 Å; the maximum deviations of 0.090 (5) and -0.117 (4) Å are found for the C10 and N1 atoms, respectively. The C13 and C14 atoms lie 0.267 (5) and -0.480 (5) Å out of this plane, respectively. In the di-anion, the Cu—I distances lie in a relatively narrow range (Table 1) and the Cu atom lies 0.1407 (6) Å above the trigonal plane defined by the iodido atoms.

In the crystal structure, the ions are almost parallel (dihedral angle between the 1,10-phenanthrolinium and CuI_3_ planes = 1.45 (4) °) with the closest interaction between them being a C—H···I contact, Table 2. The I3 atom lies over the (C4–C7,C11,C12) ring, with the I3···ring centroid distance = 3.5842 (19) Å and the Cu—I3···ring centroid angle = 101.22 (3)°.

### Experimental

5,6-Dihydrodipyrazino(1,2,3,4-lmn)-1,10-phenanthrolinium dibromide was synthesized by reacting 1,2-dibromoethane with 1,10-phenanthroline monohydrate. A methanol solution (10 ml) of the salt (0.37 g, 1 mmol) was mixed with a water/DMF (1:4) solution (10 ml) of cuprous iodide (0.19 g, 1 mmol). An excess of potassium iodide (0.83 g, 5 mmol) was added. The solution was filtered and the solvent allow to evaporate slowly to furnish dark-brown crystals of the cuprate salt.

### Refinement

H-atoms were placed in calculated positions (C—H 0.95 to 0.99 Å) and were included in the refinement in the riding model approximation with *U_iso_*(H) set to 1.2*U_eq_*(C). The anisotropic displacement ellipsoid of one of the phenanthroline C-atoms (C12) was tightly restrained to be nearly isotropic.

### Crystal data

**Table:**

(C_14_H_12_N_2_)[CuI_3_]	*F*(000) = 1192
*M**_r_* = 652.50	*D*_x_ = 2.678 Mg m^−^^3^
Monoclinic, *P*2_1_/*n*	Mo *K*α radiation, λ = 0.71073 Å
Hall symbol: -P 2yn	Cell parameters from 4486 reflections
*a* = 7.6018 (6) Å	θ = 2.9–28.1°
*b* = 15.0917 (12) Å	µ = 7.06 mm^−^^1^
*c* = 14.2776 (12) Å	*T* = 100 K
β = 98.903 (1)°	Plate, brown
*V* = 1618.2 (2) Å^3^	0.20 × 0.20 × 0.02 mm
*Z* = 4	

### Data collection

**Table:**

Bruker SMART APEX diffractometer	3701 independent reflections
Radiation source: fine-focus sealed tube	3065 reflections with *I* > 2σ(*I*)
graphite	*R*_int_ = 0.055
ω scans	θ_max_ = 27.5°, θ_min_ = 2.0°
Absorption correction: multi-scan (*SADABS*; Sheldrick, 1996)	*h* = −9→9
*T*_min_ = 0.332, *T*_max_ = 0.872	*k* = −19→19
14984 measured reflections	*l* = −18→18

### Refinement

**Table:**

Refinement on *F*^2^	Primary atom site location: structure-invariant direct methods
Least-squares matrix: full	Secondary atom site location: difference Fourier map
*R*[*F*^2^ > 2σ(*F*^2^)] = 0.028	Hydrogen site location: inferred from neighbouring sites
*wR*(*F*^2^) = 0.065	H-atom parameters constrained
*S* = 1.05	*w* = 1/[σ^2^(*F*_o_^2^) + (0.015*P*)^2^ + 1.1596*P*] where *P* = (*F*_o_^2^ + 2*F*_c_^2^)/3
3701 reflections	(Δ/σ)_max_ = 0.001
181 parameters	Δρ_max_ = 0.73 e Å^−^^3^
6 restraints	Δρ_min_ = −0.82 e Å^−^^3^

### Fractional atomic coordinates and isotropic or equivalent isotropic displacement parameters (Å^2^)

**Table:**

	*x*	*y*	*z*	*U*_iso_*/*U*_eq_	
I1	0.66182 (4)	0.44391 (2)	0.12230 (2)	0.01428 (8)	
I2	0.67359 (4)	0.43433 (2)	0.42229 (2)	0.01632 (9)	
I3	0.49833 (4)	0.19901 (2)	0.24528 (2)	0.01604 (9)	
Cu	0.62612 (8)	0.35182 (4)	0.26653 (4)	0.01510 (14)	
N1	0.1049 (5)	0.3718 (3)	0.3535 (3)	0.0130 (8)	
N2	0.1521 (5)	0.4112 (3)	0.1658 (3)	0.0119 (8)	
C1	0.0995 (6)	0.3503 (3)	0.4439 (3)	0.0160 (10)	
H1	0.1218	0.3950	0.4911	0.019*	
C2	0.0627 (6)	0.2657 (3)	0.4704 (3)	0.0169 (10)	
H2	0.0668	0.2510	0.5354	0.020*	
C3	0.0196 (6)	0.2024 (3)	0.4013 (3)	0.0165 (10)	
H3	−0.0120	0.1443	0.4182	0.020*	
C4	0.0223 (6)	0.2236 (3)	0.3056 (3)	0.0126 (9)	
C5	−0.0244 (6)	0.1603 (3)	0.2316 (3)	0.0133 (10)	
H5	−0.0615	0.1025	0.2464	0.016*	
C6	−0.0163 (6)	0.1820 (3)	0.1401 (3)	0.0140 (10)	
H6	−0.0494	0.1394	0.0916	0.017*	
C7	0.0411 (6)	0.2679 (3)	0.1155 (3)	0.0110 (9)	
C8	0.0559 (6)	0.2916 (3)	0.0224 (3)	0.0154 (10)	
H8	0.0228	0.2505	−0.0277	0.019*	
C9	0.1181 (6)	0.3739 (3)	0.0029 (3)	0.0145 (10)	
H9	0.1255	0.3906	−0.0605	0.017*	
C10	0.1702 (6)	0.4327 (3)	0.0771 (3)	0.0148 (10)	
H10	0.2190	0.4886	0.0645	0.018*	
C11	0.0871 (6)	0.3306 (3)	0.1873 (3)	0.0109 (9)	
C12	0.0725 (6)	0.3089 (3)	0.2834 (3)	0.0096 (9)	
C13	0.2245 (6)	0.4716 (3)	0.2442 (3)	0.0126 (10)	
H13A	0.2214	0.5333	0.2206	0.015*	
H13B	0.3500	0.4559	0.2678	0.015*	
C14	0.1165 (6)	0.4646 (3)	0.3240 (3)	0.0140 (10)	
H14A	0.1729	0.5006	0.3785	0.017*	
H14B	−0.0047	0.4882	0.3029	0.017*	

### Atomic displacement parameters (Å^2^)

**Table:**

	*U*^11^	*U*^22^	*U*^33^	*U*^12^	*U*^13^	*U*^23^
I1	0.01585 (16)	0.01624 (17)	0.01116 (15)	0.00100 (12)	0.00336 (12)	0.00077 (12)
I2	0.01971 (17)	0.01736 (17)	0.01211 (15)	0.00023 (13)	0.00313 (12)	−0.00156 (12)
I3	0.01528 (16)	0.01722 (17)	0.01553 (16)	−0.00182 (12)	0.00215 (12)	0.00031 (12)
Cu	0.0145 (3)	0.0173 (3)	0.0134 (3)	0.0010 (2)	0.0021 (2)	−0.0004 (2)
N1	0.0086 (19)	0.019 (2)	0.0107 (19)	0.0034 (16)	−0.0015 (15)	0.0011 (16)
N2	0.011 (2)	0.014 (2)	0.0104 (19)	−0.0011 (15)	0.0006 (16)	−0.0021 (15)
C1	0.016 (3)	0.019 (3)	0.013 (2)	0.004 (2)	0.0004 (19)	0.0003 (19)
C2	0.019 (3)	0.022 (3)	0.009 (2)	0.005 (2)	0.002 (2)	0.004 (2)
C3	0.015 (2)	0.017 (3)	0.018 (2)	0.002 (2)	0.004 (2)	0.002 (2)
C4	0.010 (2)	0.015 (2)	0.013 (2)	0.0014 (18)	0.0027 (18)	0.0013 (18)
C5	0.007 (2)	0.015 (2)	0.018 (2)	0.0005 (18)	0.0022 (18)	0.0038 (19)
C6	0.011 (2)	0.014 (2)	0.016 (2)	0.0016 (18)	0.0020 (19)	−0.0015 (19)
C7	0.009 (2)	0.013 (2)	0.011 (2)	0.0007 (18)	0.0011 (17)	−0.0016 (18)
C8	0.014 (2)	0.017 (3)	0.015 (2)	0.0033 (19)	0.0025 (19)	−0.0017 (19)
C9	0.018 (3)	0.018 (3)	0.009 (2)	0.000 (2)	0.0046 (19)	0.0003 (19)
C10	0.012 (2)	0.020 (3)	0.012 (2)	0.0003 (19)	0.0013 (19)	0.004 (2)
C11	0.008 (2)	0.013 (2)	0.012 (2)	0.0025 (17)	0.0009 (17)	0.0034 (18)
C12	0.0033 (19)	0.013 (2)	0.012 (2)	0.0025 (16)	0.0008 (16)	−0.0009 (17)
C13	0.014 (2)	0.011 (2)	0.012 (2)	−0.0016 (18)	−0.0024 (18)	−0.0007 (18)
C14	0.017 (3)	0.010 (2)	0.016 (2)	0.0032 (19)	0.006 (2)	−0.0020 (18)

### Geometric parameters (Å, °)

**Table:**

Cu—I1	2.5336 (7)	C5—C6	1.357 (6)
Cu—I2	2.5254 (7)	C5—H5	0.9500
Cu—I3	2.5025 (7)	C6—C7	1.429 (6)
N1—C1	1.337 (6)	C6—H6	0.9500
N1—C12	1.375 (6)	C7—C8	1.398 (6)
N1—C14	1.470 (6)	C7—C11	1.399 (6)
N2—C10	1.336 (6)	C8—C9	1.373 (7)
N2—C11	1.366 (6)	C8—H8	0.9500
N2—C13	1.481 (6)	C9—C10	1.391 (6)
C1—C2	1.374 (7)	C9—H9	0.9500
C1—H1	0.9500	C10—H10	0.9500
C2—C3	1.376 (7)	C11—C12	1.431 (6)
C2—H2	0.9500	C13—C14	1.507 (6)
C3—C4	1.406 (6)	C13—H13A	0.9900
C3—H3	0.9500	C13—H13B	0.9900
C4—C12	1.394 (6)	C14—H14A	0.9900
C4—C5	1.428 (7)	C14—H14B	0.9900
			
I3—Cu—I2	124.13 (3)	C11—C7—C6	118.9 (4)
I3—Cu—I1	119.69 (2)	C9—C8—C7	120.3 (4)
I2—Cu—I1	115.25 (3)	C9—C8—H8	119.9
C1—N1—C12	120.5 (4)	C7—C8—H8	119.9
C1—N1—C14	121.3 (4)	C8—C9—C10	119.3 (4)
C12—N1—C14	117.5 (4)	C8—C9—H9	120.4
C10—N2—C11	121.5 (4)	C10—C9—H9	120.4
C10—N2—C13	119.1 (4)	N2—C10—C9	120.6 (4)
C11—N2—C13	118.9 (4)	N2—C10—H10	119.7
N1—C1—C2	121.9 (5)	C9—C10—H10	119.7
N1—C1—H1	119.0	N2—C11—C7	119.7 (4)
C2—C1—H1	119.0	N2—C11—C12	120.2 (4)
C1—C2—C3	119.0 (4)	C7—C11—C12	120.0 (4)
C1—C2—H2	120.5	N1—C12—C4	119.8 (4)
C3—C2—H2	120.5	N1—C12—C11	120.6 (4)
C2—C3—C4	120.0 (5)	C4—C12—C11	119.7 (4)
C2—C3—H3	120.0	N2—C13—C14	110.3 (4)
C4—C3—H3	120.0	N2—C13—H13A	109.6
C12—C4—C3	118.5 (4)	C14—C13—H13A	109.6
C12—C4—C5	119.6 (4)	N2—C13—H13B	109.6
C3—C4—C5	121.8 (4)	C14—C13—H13B	109.6
C6—C5—C4	120.5 (4)	H13A—C13—H13B	108.1
C6—C5—H5	119.8	N1—C14—C13	110.3 (4)
C4—C5—H5	119.8	N1—C14—H14A	109.6
C5—C6—C7	121.2 (4)	C13—C14—H14A	109.6
C5—C6—H6	119.4	N1—C14—H14B	109.6
C7—C6—H6	119.4	C13—C14—H14B	109.6
C8—C7—C11	118.5 (4)	H14A—C14—H14B	108.1
C8—C7—C6	122.6 (4)		
			
C12—N1—C1—C2	0.3 (7)	C8—C7—C11—N2	−2.8 (7)
C14—N1—C1—C2	170.3 (4)	C6—C7—C11—N2	176.2 (4)
N1—C1—C2—C3	−4.0 (7)	C8—C7—C11—C12	179.6 (4)
C1—C2—C3—C4	3.1 (7)	C6—C7—C11—C12	−1.4 (7)
C2—C3—C4—C12	1.2 (7)	C1—N1—C12—C4	4.2 (6)
C2—C3—C4—C5	−178.9 (4)	C14—N1—C12—C4	−166.2 (4)
C12—C4—C5—C6	1.5 (7)	C1—N1—C12—C11	−176.8 (4)
C3—C4—C5—C6	−178.4 (4)	C14—N1—C12—C11	12.8 (6)
C4—C5—C6—C7	0.8 (7)	C3—C4—C12—N1	−4.8 (6)
C5—C6—C7—C8	178.1 (4)	C5—C4—C12—N1	175.2 (4)
C5—C6—C7—C11	−0.9 (7)	C3—C4—C12—C11	176.1 (4)
C11—C7—C8—C9	1.5 (7)	C5—C4—C12—C11	−3.8 (6)
C6—C7—C8—C9	−177.6 (4)	N2—C11—C12—N1	7.2 (6)
C7—C8—C9—C10	1.5 (7)	C7—C11—C12—N1	−175.3 (4)
C11—N2—C10—C9	1.8 (7)	N2—C11—C12—C4	−173.9 (4)
C13—N2—C10—C9	174.2 (4)	C7—C11—C12—C4	3.7 (6)
C8—C9—C10—N2	−3.2 (7)	C10—N2—C13—C14	150.5 (4)
C10—N2—C11—C7	1.2 (7)	C11—N2—C13—C14	−36.9 (6)
C13—N2—C11—C7	−171.2 (4)	C1—N1—C14—C13	146.4 (4)
C10—N2—C11—C12	178.8 (4)	C12—N1—C14—C13	−43.3 (5)
C13—N2—C11—C12	6.4 (6)	N2—C13—C14—N1	53.7 (5)

### Hydrogen-bond geometry (Å, °)

**Table:**

*D*—H···*A*	*D*—H	H···*A*	*D*···*A*	*D*—H···*A*
C13—H13b···I2	0.99	3.06	3.969 (4)	154
